# Bullatacin triggers immunogenic cell death of colon cancer cells by activating endoplasmic reticulum chaperones

**DOI:** 10.1186/s12950-021-00289-1

**Published:** 2021-06-10

**Authors:** Fangtian Fan, Peiliang Shen, Yue Ma, Wangbo Ma, Hongyan Wu, Hao Liu, Qing An

**Affiliations:** 1 Anhui Engineering Technology Research Center of Biochemical Pharmaceuticals, School of Pharmacy, Bengbu Medical College, 2600 Donghai Avenue, Bengbu, 233003 Anhui China; 2 School of Pharmacy, Nanjing University of Chinese Medicine, Nanjing, 210023 China; 3 Institute of Biomedical Technology, Jiangsu Vocational College of Medicine, No.283 Jiefang South Road, Yancheng, 224005 China; 4 Jiangsu Cancer Hospital & Jiangsu Institute of Cancer Research & The Affiliated Cancer Hospital of Nanjing Medical University, Nanjing, 210009 China

**Keywords:** Bullatacin, ICD, Damage-associated molecular patterns (DAMPs), Immunotherapy, Phagocytosis

## Abstract

**Background:**

It is well accepted that the immune system efficiently contributes to positive outcomes of chemotherapeutic cancer treatment by activating immunogenic cell death (ICD). However, only a limited number of ICD-inducing compounds are well characterized at present; therefore, identification of novel ICD inducers is urgently needed for cancer drug discovery, and the need is becoming increasingly urgent.

**Methods:**

Herein, we assessed the antitumour activity of bullatacin by MTS assay and apoptosis assay. ICD biomarkers, such as calreticulin (CRT), high-mobility group protein B1 (HMGB-1), heat shock protein (HSP)70, HSP90 and ATP, were assessed by Western blotting, ELISA and flow cytometry. Western blot and qPCR assays were performed to explore the underlying mechanisms of bullatacin-induced ICD. Flow cytometry was used to detect macrophage phagocytosis.

**Results:**

First, bullatacin induced apoptosis in both SW480 cells and HT-29 cells in a time-dependent manner at 10 nM, as assessed by flow cytometry. Moreover, Western blot and flow cytometry assays showed that CRT and HSP90 (biomarkers of early ICD) significantly accumulated on the cell membrane surface after approximately 6 h of treatment with bullatacin. In addition, ELISAs and Western blot assays showed that the second set of hallmarks required for ICD (HMGB1, HSP70 and HSP90) were released in the conditioned media of both SW480 and HT-29 cells after 36 h of treatment. Furthermore, qPCR and Western blot assays indicated that bullatacin triggered ICD via activation of the endoplasmic reticulum stress (ERS) signalling pathway. Finally, bullatacin promoted macrophage phagocytosis.

**Conclusion:**

This study documents that bullatacin, a novel ICD inducer, triggers immunogenic tumour cell death by activating ERS even at a relatively low concentration in vitro.

## Introduction

Immunotherapies for the treatment of solid tumours that block immunoregulatory checkpoints, namely, PD-1/PDL1 and cytotoxic T lymphocyte-associated protein 4 (CTLA-4), have paved the way for therapeutic breakthroughs in clinical practice [1, 2]. However, such strategies do not yield long-lasting clinical outcomes, and immunotherapy remains a clinical challenge with a success rate of less than 20%. Recently, it has been documented that low immunogenicity of tumours is a key reason for immunotherapy failure [3]. Therefore, strategies enhancing tumour immunogenicity can be applied to improve the efficacy of cancer immunotherapy.

Accumulating evidence shows that the anticancer potential of the immune response can be activated through modulation of the immunogenicity of cancer cells dying in a regulated cell death process called immunogenic cell death (ICD) [4–7]. The immunogenic characteristics of ICD are mediated mainly by dead-cell antigens, particularly those derived from damage-associated molecular patterns (DAMPs), including surface-exposed calreticulin (CRT), heat shock protein (HSP), secreted ATP and released high-mobility group protein B1 (HMGB1) [7, 8]. Most DAMPs can be recognized by pattern recognition receptors (PRRs), which alert antigen-presenting cells (APCs) to break them down into small fragments that serve as antigens for priming and expansion of human cytotoxic T lymphocytes (CTLs) [4, 7].

Evidence from laboratory studies and clinical trials has indicated that conventional cytotoxic anticancer drugs, such as cisplatin and anthracycline, that induce immunogenic death can significantly improve the immune responses mediated by checkpoint inhibitors [6, 7]. However, so far, only a limited number of anticancer agents triggering ICD have been found, including natural compounds (doxorubicin and mitoxantrone) and synthetic molecules (oxaliplatin and cyclophosphamide) [9, 10]. Therefore, the identification of more new ICD inducers is becoming increasingly urgent.

Bullatacin is a natural compound isolated from plants in the genera of the Annonaceae family that exhibits significant antitumour activity [11–13]. Notably, bullatacin has shown cytotoxicity towards various tumour cells even at concentrations in the nanomolar range, including human colon cancer cells, lung cancer cells and breast cancer cells [14]. Moreover, previous studies have demonstrated that bullatacin exhibits strong antitumour effects in vivo with low toxicity [12]; however, the underlying mechanism remains unclear. Considering that bullatacin can induce apoptosis, we hypothesized that bullatacin is likely to induce immunogenic death of tumour cells. In the present study, we investigated the inducing effect of bullatacin on immunogenic death of human colon cancer cells and the underlying molecular mechanisms.

## Results

### Bullatacin induces apoptosis in colon cancer cells

**Fig. 1:**
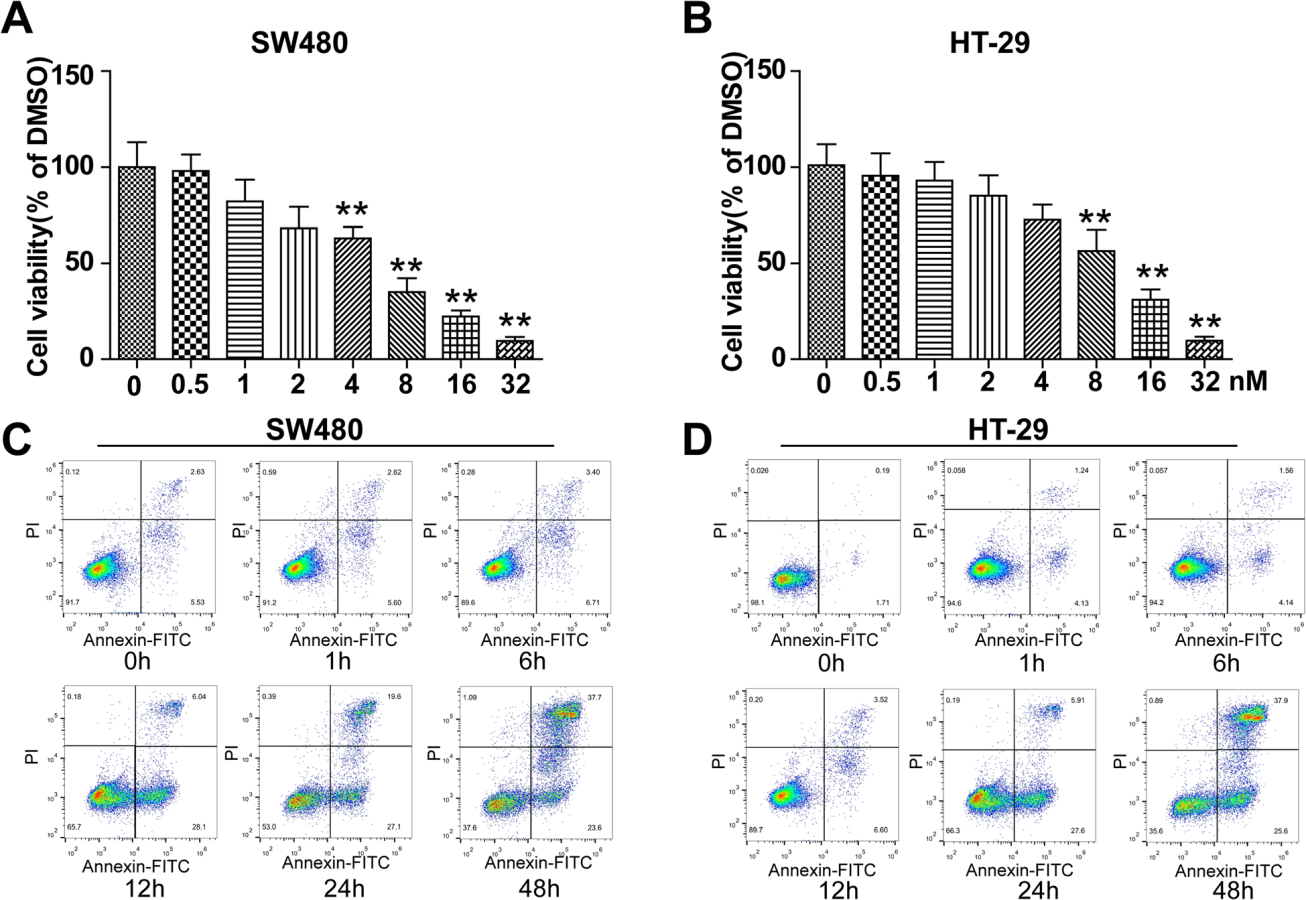
Bullatacin induces apoptosis in colon cancer cells. **A** CCK-8 assay of the cell viability of SW480 cells treated with bullatacin for 24 h. Significance: **P < 0.01 versus control. **B** CCK-8 assay of the cell viability of HT-29 cells treated with bullatacin for 24 h. Significance: **P < 0.01 versus control. **C** Flow cytometric analysis of the time-dependent apoptosis of SW480 cells treated with bullatacin (10 nM). **D** Flow cytometric analysis of the time-dependent apoptosis of HT-29 cells treated with bullatacin (10 nM)

Colon cancer cells, including SW480 and HT-29 cells, were incubated with different concentrations of bullatacin for 48 h, and cell viability was analysed by CCK-8 assay. The results showed that bullatacin dose-dependently reduced cell viability in the two cell lines, and the IC50 values were approximately 10 nM and 7 nM, respectively (Fig. 1A, B), consistent with a previous study [14]. Given that bullatacin induces hepatocarcinoma cell line apoptosis [12], to further elucidate whether the cytotoxic effects of bullatacin were due to apoptosis, we next examined whether bullatacin (10 nM) could induce an apoptotic response in colon cancer cells via flow cytometry. We found that bullatacin induced both SW480 cell and HT-29 cell apoptosis in a time-dependent manner at the indicated concentrations (Fig. 1C, D).

### Bullatacin induces the expression of CRT and HSP90 on the cell membrane surfaces of early apoptotic cells

**Fig. 2:**
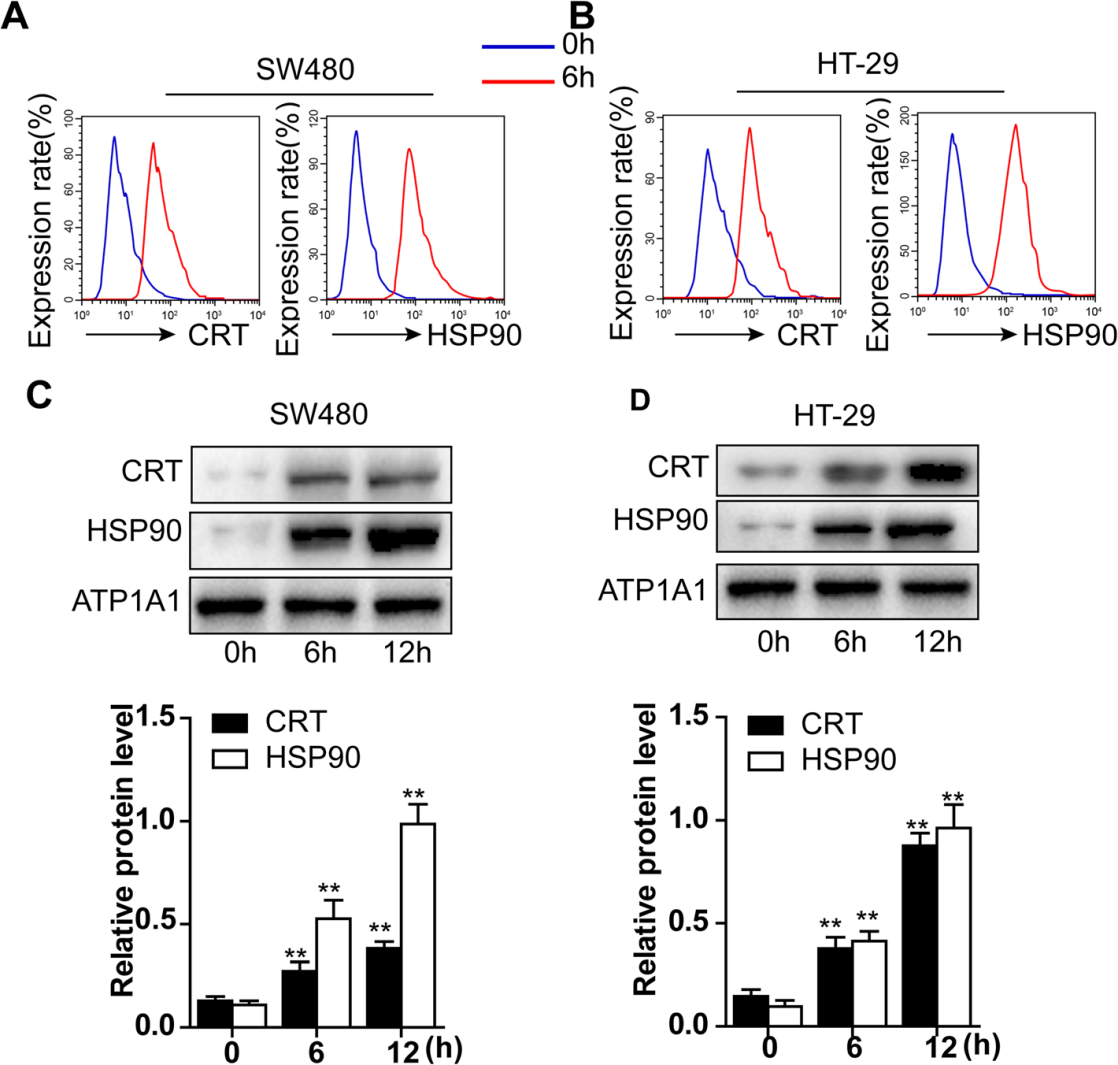
Bullatacin induces the expression of CRT and HSP90 on the cell membrane surface in early apoptotic cells. **A**, **B** Flow cytometric analyses of the protein expression of CRT and HSP90 in SW480 and HT-29 cells treated with bullatacin for 12 h. **C**, **D** Western blot analyses of the cell membrane protein expression of CRT and HSP90 in SW480 and HT-29 cells treated with bullatacin for the indicated times. The relative protein expression of CRT and HSP90 was evaluated by quantifying the greyscale values with ImageJ. Significance: *P < 0.05 versus control, **P < 0.01 versus control

As a previous study has reported that bullatacin (25 μg/kg) significantly inhibits ovarian cancer with low toxicity in vivo [15], we hypothesized that bullatacin might induce tumour ICD and investigated whether bullatacin could induce the ICD response in HT-29 and SW480 cells. CRT exposure is the result of relocation of ER-resident CRT to the plasma membrane [16]. Surface CRT and HSP90 both act as “eat me” signals, triggering APC-mediated dead-cell antigen uptake, a crucial event for priming of the innate immune response [17]. Therefore, we used flow cytometry to assess the membrane expression of CRT and HSP90, which are biomarkers of early ICD. The results showed that CRT and HSP90 significantly accumulated on the cell membrane surface after approximately 6 h of treatment with bullatacin (Fig. 2A, B). Moreover, we also used Western blotting to confirm the expression of CRT and HSP90 on the surface of the cell membrane, and the results were consistent with those of the flow cytometry assay (Fig. 2C, D).

### Bullatacin induces ATP release in early apoptotic cells

**Fig. 3:**
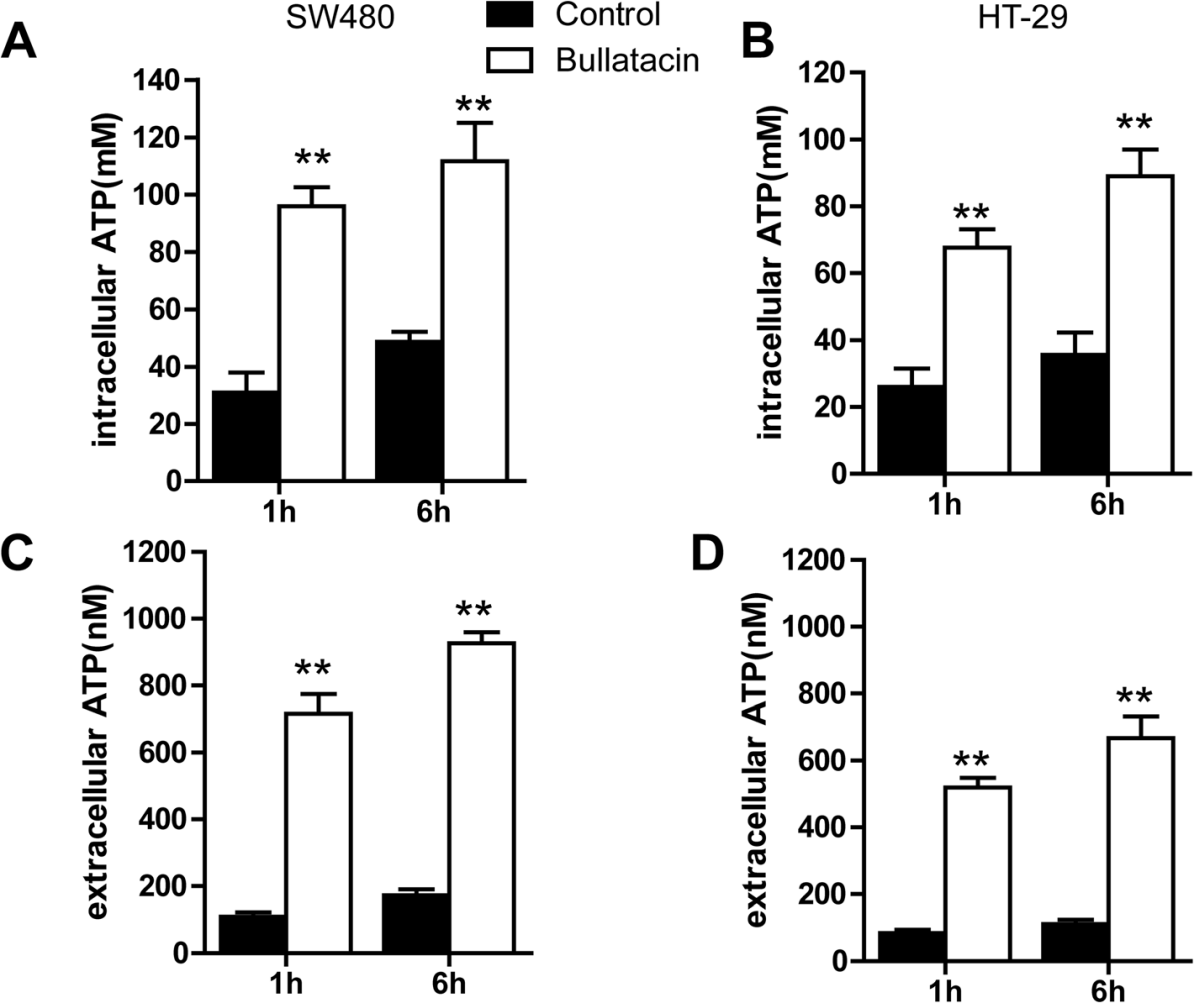
Bullatacin induces ATP release in early apoptotic cells. **A**–**D** An ATP Assay Kit was used to analyse intra- and extracellular ATP levels at the indicated times in SW480 and HT-29 cells. Significance:**P < 0.01 versus control

Since secretion of ATP is mandatory for the induction of specific antitumour immunity [18, 19], extracellular ATP acts both as a “find me” signal and as an activator of the NOD-like receptor family pyridine domain containing-3 (NLRP3) inflammasome, thereby stimulating both the recruitment and activation of APCs required for adequate polarization of cytotoxic T lymphocytes. Therefore, we investigated whether bullatacin could induce the release of ATP in pre- and early apoptotic cells. The results showed that both intracellular and extracellular ATP levels were significantly upregulated after treatment with bullatacin for 1 h (10 nM) (Fig. 3A–D) in SW480 and HT-29 cell lines. Our results indicate that bullatacin induces ATP release before the onset of apoptosis.

### Bullatacin promotes the release of HMGB1, HSP90 and HSP70 in late apoptotic cells

**Fig. 4:**
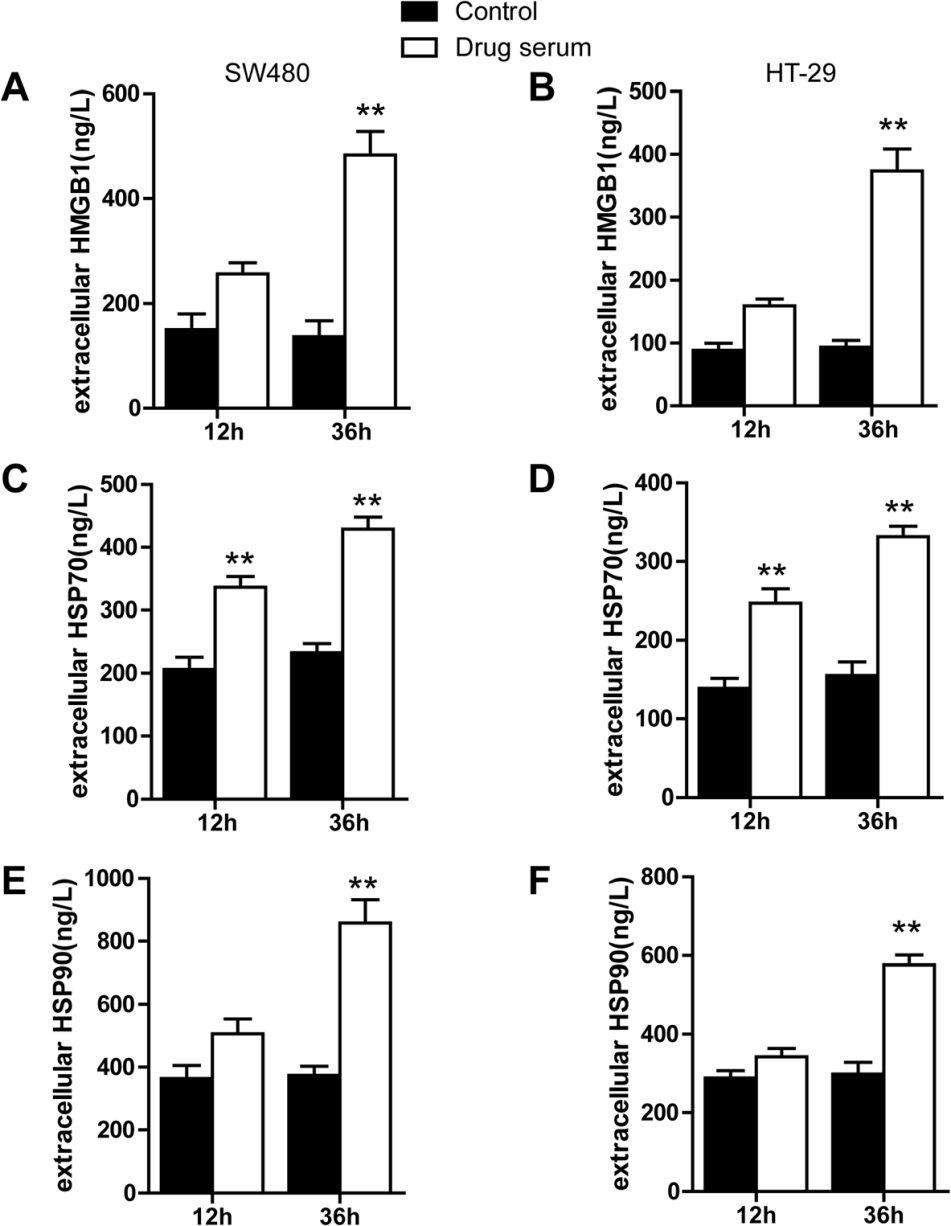
Bullatacin promotes the release of HMGB1, HSP90 and HSP70 in late apoptotic cells. **A** ELISA was used to analyse the concentration of HMGB1 in the supernatant of SW480 and HT-29 cells treated with bullatacin for the indicated times. Significance:**P < 0.01 versus control. **B** ELISA was used to analyse the concentration of HSP90 in the supernatant of SW480 and HT-29 cells treated with bullatacin for the indicated times. Significance: **P < 0.01 versus control. **C** ELISA was used to analyse the concentration of HSP70 in the supernatant of SW480 and HT-29 cells treated with bullatacin for the indicated times. Significance: **P < 0.01 versus control

HMGB1 release is mandatory for ICD because HMGB1, by interacting with APCs, triggers signalling pathways that allow the antigen to be trafficked towards the antigen-presenting compartment, thereby leading to optimal tumour antigen processing and cross-presentation to T cells [9]. Thus, we next investigated whether bullatacin-induced apoptosis was associated with the late apoptotic extracellular passive release of HMGB1, HSP70 and HSP90, the second set of hallmarks required for ICD. As shown in Fig. 4A–C, HMGB1, HSP70 and HSP90 were released in the conditioned media of both SW480 and HT-29 cells after 36 h of treatment.

### Bullatacin promotes macrophage phagocytosis

**Fig. 5:**
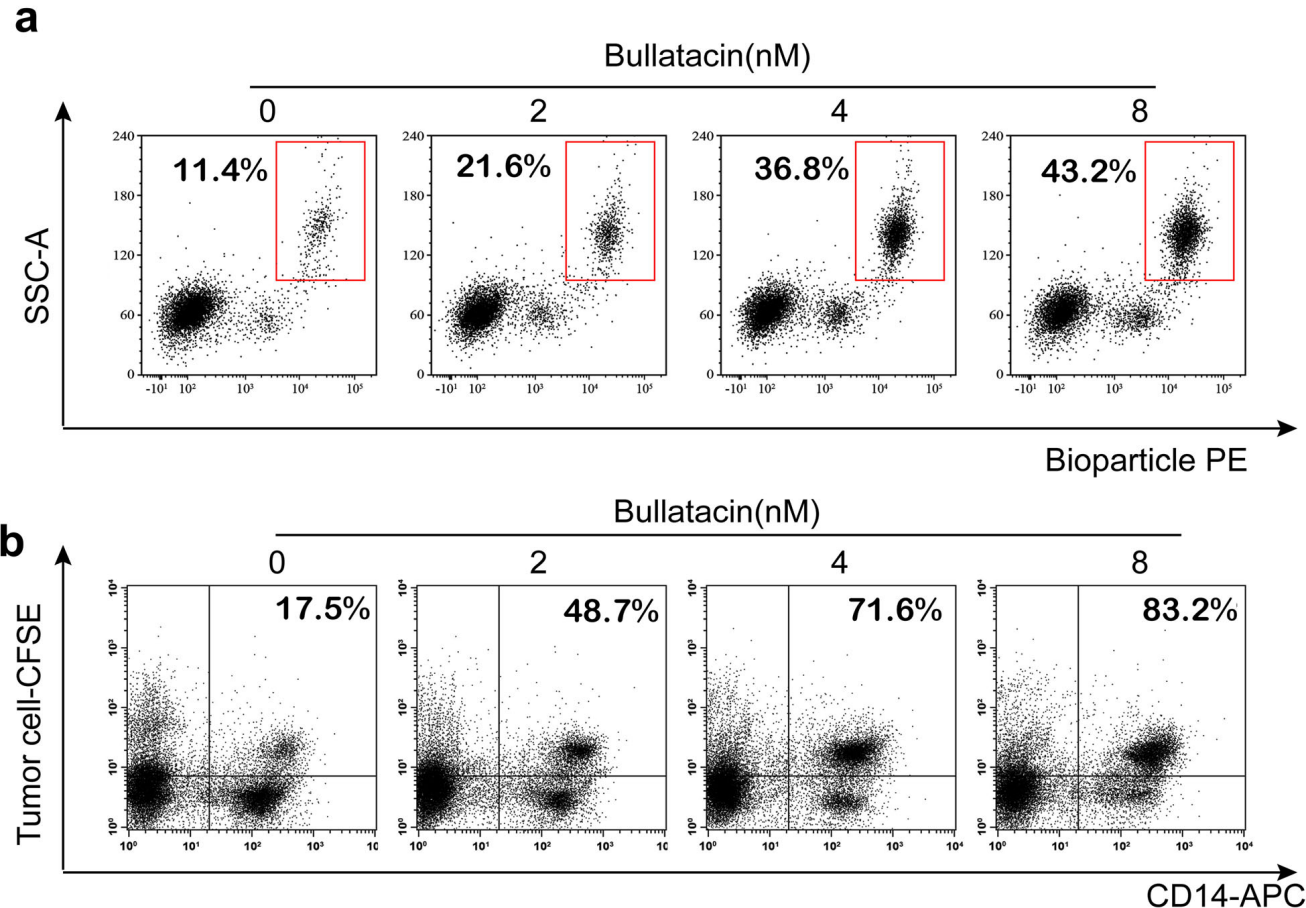
**a** The phagocytosis of PE labeled fluorescent microspheres by macrophages was analyzed by flow cytometry, the double fluorescence in the upper right corner indicates that macrophages phagocytosed fluorescent microspheres. **b** The phagocytosis of CFSE labeled tumor cells by macrophages was analyzed by flow cytometry,the double fluorescence in the upper right corner indicates that macrophages phagocytize fluorescent tumor cell. Significance: **P <0.01 versus control

It has been reported that ICD biomarkers, including CRT, HMGB1 and ATP, can trigger phagocytosis [20]. The above results have demonstrated that bullatacin could induce the release of ICD biomarkers, but whether it actually triggers phagocytosis remains to be investigated. Therefore, macrophage phagocytosis of microspheres or tumour cells after bullatacin treatment was evaluated in vitro. The flow cytometry scatter plot showed that bullatacin treatment enhanced phagocytosis of both microspheres and tumour cells by macrophages (Fig. 5A, B). These results indicate the medium from tumour cell treated with bullatacin did activate macrophages and enhance their phagocytic function.

### Bullatacin triggers ICD via activation of the ERS signalling pathway

**Fig. 6:**
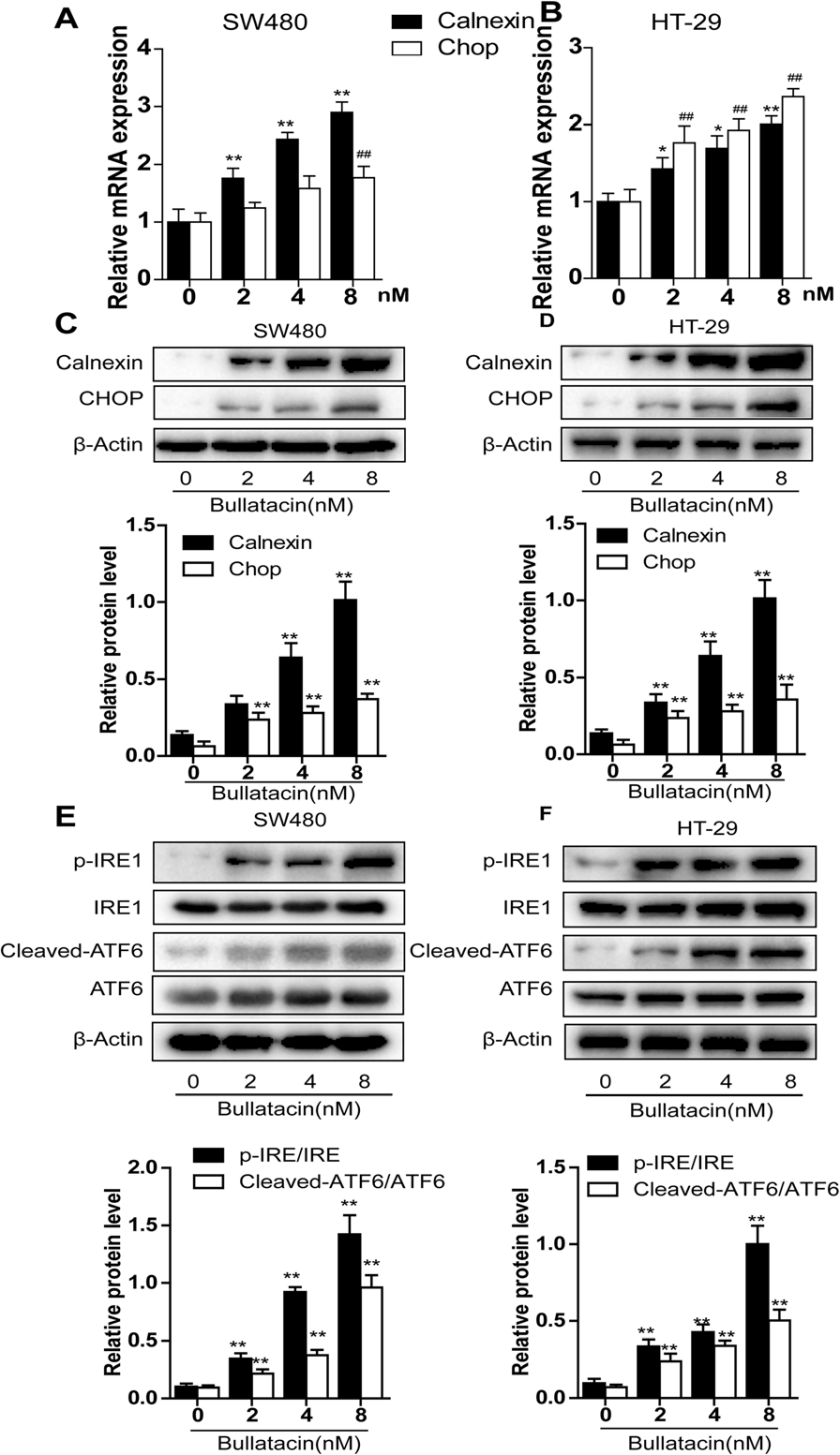
Bullatacin triggers ICD via activation of the ERS signalling pathway. **A**, **B** Real-time PCR analyses of the mRNA expression of calnexin and CHOP in two colon cancer cell lines treated with bullatacin at the indicated concentrations. Significance: **P < 0.01 versus control. **C**, **D** Western blot analyses of the cell membrane protein expression of CRT and HSP90 in SW480 and HT-29 cells treated with bullatacin for the indicated times. The relative protein expression of CRT and HSP90 was evaluated by quantifying the greyscale values with ImageJ. Significance: **P < 0.01 versus control. Western blot analyses of the protein expression of IRE1, pIRE1, cleaved-ATF6 and ATF6 in SW480 and HT-29 cells treated with bullatacin for 12 h. The relative protein expression of IRE1, pIRE1, cleaved-ATF6 and ATF6 was evaluated by quantifying the greyscale values with ImageJ. Significance: **P < 0.01 versus control

Recently, central roles for the endoplasmic reticulum (ER) stress response in all scenarios of ICD have been described [9, 21, 22]. To this end, we next elucidated the molecular pathways relating to ER stress that mediated the bullatacin-induced promotion of ICD. We detected the expression of calnexin, an endoplasmic reticulum chaperone, and the transcription factor C/EBP homologous protein (CHOP), a major marker of prolonged ERS. The data demonstrated that 10 nM bullatacin significantly upregulated the mRNA expression of calnexin and CHOP in two colon cancer cell lines (Fig. 6A, B). Likewise, we obtained consistent results in Western blotting analyses (Fig. 6C, D). Furthermore, upon stress exposure, the UPR is initiated by the activation of three endoplasmic reticulum transmembrane proteins: PERK, IRE1 and ATF6 [23, 24]. Our subsequent examinations showed that bullatacin promoted the phosphorylation of PERK and IRE1 and stimulated the cleavage of ATF6 in the two cell lines (Fig. 6E, F). Collectively, these data indicated that bullatacin selectively activated the ERS pathway in colon cancer cells.

## Discussion

It has been documented that various members of the plant family Annonaceae produce a cluster of bioactive secondary metabolites known as Annonaceous acetogenins (AAs). AAs have attracted researchers’ attention in recent years due to their unique structures and wide range of biological activities [12]. Since the initial discovery of uvaricin in 1982, over 300 of these natural AAs have been obtained by using a murine leukaemia antitumour system [25]. They have been shown to be cytotoxic, pesticidal, antimalarial, antiparasitic and antimicrobial and to have in vivo antitumour effects [26]. Bullatacin, isolated from the fruit of *Annona atemoya*, is one of the most potentially effective antitumour AAs [11, 27]. According to the data of the National Cancer Institute (NCI), it is effective for lung cancer, liver cancer, breast cancer, bladder cancer, cervical cancer and lymphoma [14, 15]. Moreover, the antitumour effect of bullatacin has been reported to involve mainly induction of apoptosis. Chiu et al. found that bullatacin can induce apoptosis of HCC cells in a time- and dose-dependent manner and that its mechanism is related to changes in the levels of cAMP and cGMP [12]. Further studies have shown that the pathway is mitochondria dependent and is related mainly to activation of caspase-9 [16]. Considering that bullatacin can induce apoptosis and exhibits low toxicity in vivo, in this study, we investigated whether bullatacin can induce immunogenic death of tumour cells.

When cells undergo immunogenic death, the cells produce large numbers of DAMPs (including the endoplasmic reticulum molecular chaperone protein calreticulin, which is transferred from the endoplasmic reticulum to the cell membrane; ATP, which is released; HMGB1, which upregulated; etc.), and these DAMPs can interact with pattern recognition receptors on immune cells and have strong immune stimulation effects [6, 7]. Our results suggest that the endoplasmic reticulum molecular chaperone calreticulin and HSP90 are transferred to the cell membrane after treatment with bullatacin. In addition, ATP and HMGB1 protein levels in both tumour cells and culture supernatant are significantly increased upon treatment with bullatacin. These results jointly indicate that bullatacin promotes the immunogenic death of cells by increasing the expression of DAMPs in tumour cells, which may contribute to its high antitumour activity and low toxicity to normal tissue.

Endoplasmic reticulum stress is an important cause of immunogenic death of tumour cells [22]. In the endoplasmic reticulum, nascent proteins are folded with the assistance of ER chaperones. However, the accumulation of unfolded and/or misfolded proteins in the endoplasmic reticulum lumen, creating the condition of ERS, results in activation of the unfolded protein response (UPR) [23]. Under severe and prolonged ER stress conditions, the UPR is unable to restore normal cellular function. Calnexin and CHOP are two important markers of ERS [24]. Our results showed that these two molecules were concomitantly upregulated by bullatacin, strongly indicating activation of the ERS pathway. Furthermore, upon ERS, the sensors PERK, IRE1 and ATF6 dissociate from chaperones and initiate three arms of signalling events, resulting in increased expression of genes critical for overcoming ERS, including transcription factors and molecular chaperones [21]. Therefore, we detected these key factors and found that they were all activated in bullatacin-treated cancer cells. These results collectively demonstrate that bullatacin-induced immunogenic death of tumour cells may be related to increased endoplasmic reticulum stress in tumour cells.

In summary, this study documents that bullatacin, a novel ICD inducer, triggers immunogenic tumour cell death by activating endoplasmic reticulum stress even at a low concentration in vitro. However, whether it can activate tumour immune function needs to be validated in vivo.

## Conclusions

In conclusion, we explored that bullatacin triggers immunogenic tumour cell death by activating ERS. Bullatacin, a novel ICD inducer, which may be excellent strategies enhancing tumour immunogenicity can be applied to improve the efficacy of cancer immunotherapy.

## Methods

### Cell culture

SW480 and HT-29 cells were purchased from the Cell Bank of the Chinese Academy of Sciences (Shanghai, China). The cells were cultured in Dulbecco’s modified Eagle’s medium (Invitrogen, Grand Island, NY) supplemented with 100 U/ml penicillin, 10% foetal bovine serum (Gibco, Grand Island, NY) and 100 mg/ml streptomycin and finally grown at 37°C in a humidified atmosphere comprising 95% air and 5% CO_2_.

### Cell viability assay

SW480 and HT-29 cells were seeded into 96-well plates with 8 × 10^3^ cells/well in 0.1 ml of medium and cultured for 24 h. Then, the cells were pretreated with concentrations of 0.5, 1, 2, 4, 8, 16 and 32 nM bullatacin (Wuhan ChemNorm Biotech Co., Ltd. Wuhan, China) or an equal volume of DMSO control for 24 h. Cell viability was measured by Cell Counting Kit-8 (CCK-8) (Dojindo Chemical Technology Co., Ltd., Shanghai, China) assay according to the manufacturer’s instructions. The absorbance of CCK-8 was obtained at 450 nm using a microplate reader (BioTek Instruments, Inc.).

### Quantification of apoptosis

SW480 and HT-29 cells were seeded into 6-well plates at 8 × 10^5^ cells/well in 0.4 ml of medium and cultured for 24 h. Then, the cells were pretreated with concentrations of 0.5, 1, 2, 4, 8, 16 and 32 nM bullatacin for 0, 1, 6, 12, 24 and 48 h. After the treatment, all groups of apoptotic cells were quantitated with two-colour analysis of FITC-labelled Annexin V binding and PI uptake using an Annexin V Apoptosis Detection Kit (KeyGEN Biotech Co., Ltd., Nanjing, China) by flow cytometry according to the manufacturer’s directions.

### Calreticulin cell surface expression and HSP90 assays

SW480 and HT-29 cells were seeded into 6-well plates at 8 × 10^5^ cells/well in 0.4 ml of medium and cultured for 24 h. Then, the cells were subjected to various treatments, and after that, the culture medium was removed. The cells were collected and washed in PBS 3 times. The cells were collected in a 1.5-ml EP tube, and 3–5 μl of anti-calreticulin (CRT)-FITC and anti-hsp90-PE fluorescent antibodies or IgG fluorescent antibodies were added. The cells were incubated with the primary antibodies at room temperature for 30 min. Then, the cells were washed 3 times and subjected to flow cytometry according to the manufacturer’s directions.

### ELISA

SW480 and HT-29 cells were seeded into 12-well plates at 8 × 10^4^ cells/well in 0.3 ml of medium and cultured for 24 h. Then, the cells were subjected to various treatments, and after that, the culture medium was collected, and the supernatant was obtained by centrifugation (3000×*g* for 10 min). The levels of HMGB-1, HSP 70 and HSP 90 in the supernatant were assayed with commercial enzyme-linked immunosorbent assay (ELISA) kits according to the manufacturers’ manuals.

### ATP assay

SW480 and HT-29 cells were seeded into 6-well plates at 8 × 10^5^ cells/well in 0.4 ml of medium and cultured for 24 h. Then, the cells were subjected to various treatments, and after that, the supernatant was assayed for ATP production using an ATP Assay Kit according to the manufacturer’s instructions.

### Immunoblotting

Total lysates from treated cells were prepared with RIPA buffer. The lysates were sonicated for 15 s and centrifuged at 14,000 rpm for 10 min at 4°C. The protein concentration was determined by a bicinchoninic acid assay with BSA as a standard. Equivalent amounts of protein (20 μg/lane) were separated on 8–12% SDS polyacrylamide gels and transferred to polyvinylidene difluoride membranes (Millipore, Bedford, MA, USA). The membranes were incubated with PBS containing 0.05% Tween 20 and 5% nonfat dry milk to block nonspecific binding. The membranes were then incubated with primary antibodies (against calnexin, CHOP, PERK, p-PERK, IRE1, p-IRE1, ATF6 and β-actin) purchased from Cell Signaling Technology (Boston, USA) and then with appropriate secondary antibodies conjugated to horseradish peroxidase. The immunoreactive bands were visualized by using Renaissance Chemiluminescence Reagent (Perkin-Elmer Life Science, Boston, MA, USA).

### Real-time PCR

Total RNA was isolated from SW480 and HT-29 cells using TRIzol, and its purity was confirmed by assessment of the A260/A280 ratio. Then, the mRNA was reverse-transcribed into first-strand cDNA using an All-in-One™ First Strand cDNA Synthesis Kit. Following reverse transcription, All-in-One qPCR Primer (2 μM) and primers for the glyceraldehyde 3-phosphate dehydrogenase gene (Gapdh; Qiagen, QT01658692) were used to quantify the mRNA expression levels of various genes using an ABI 7300HT Real-time PCR System (Applied Biosystems, Foster City, CA, USA). Amplification was performed using RT2 SYBR Green ROX qPCR Mastermix under the following conditions: 95°C for 10 min followed by 40 cycles of 95°C for 10 s, 60°C for 20 s and 72°C for 15 s. Immediately following the amplification step, a single cycle of a dissociation (melting) curve program was run with steps of 95°C for 15 s, 60°C for 20 s, 95°C for 15 s and 60°C for 15 s. This cycle was followed by a melting curve analysis; the baseline and cycle threshold (Ct) values were automatically determined using ABI 7300HT software. The relative mRNA expression was calculated using the following formula: ΔΔC expression = 2^−ΔΔCt^, where ΔΔCt = ΔCt (modulated group) − ΔCt (control group), ΔCt = Ct (target gene) − Ct (β-actin) and Ct = the cycle at which the threshold was reached. The relative mRNA expression in the normal control group was set to an arbitrary unit of 1, and the gene expression in the modulated groups is presented as the fold change compared to that in the control group after normalization to β-actin expression.

### Phagocytosis assay

For qualitative analysis, macrophages stained with an APC-conjugated anti-CD14 antibody were seeded into 6-well plates with 8 × 10^4^ cells/well in 0.4 ml of medium and cultured for 18 h. Then, the cells were pretreated with concentrations of 0, 2, 4 and 8 nM bullatacin for 24 h. After treatment, the PE-conjugated BioParticles or 4 × 10^4^ HT-29 cells labelled with CFSE were added to 6-well plates and incubated for 24 h [28]. After the BioParticles or tumour cells were phagocytosed by macrophage, the fluorescence in the macrophage was measured by flow cytometry.

### Statistical analysis

Statistical analysis was performed using SPSS 10.0 software, and the results are expressed as the mean ± SD (standard deviation). The experimental data were assessed for a Gaussian distribution. Two-tailed unpaired Student’s t tests were applied for comparison of two normally distributed groups; comparisons between more than two normally distributed groups were made by one-way ANOVA followed by pairwise multiple comparison (Student-Newman-Keuls method, q-test). Differences were considered statistically significant at *P* < 0.05.

## Data Availability

All data used during the current study are available from the corresponding authors on reasonable request.
